# Business models and organizational roles of data spaces: A framework for value creation in data ecosystems

**DOI:** 10.1016/j.dib.2025.111795

**Published:** 2025-06-18

**Authors:** Jens Gessler, Maiara Rosa Cencic, Christian Metzner, Horst Wieker, Kai Lindow, Wolfgang H. Schulz

**Affiliations:** a Chair of Mobility, Trade and Logistics, Zeppelin University, Am Seemooser Horn 20, 88045, Friedrichshafen, Germany; b Fraunhofer Institute for Production Systems and Design Technology IPK, Pascalstrasse 8-9, 10587, Berlin, Germany; c Saarland University of Applied Sciences, Research Group Traffic Telematics, Goebenstrasse 40, 66117 Saarbrücken, Germany

**Keywords:** Data ecosystems, Digital platforms, Gaia-X, Business innovation, Ecosystem governance, Digital sovereignty

## Abstract

Data spaces and ecosystems provide decentralized digital infrastructures enabling secure, sovereign, and inter-organizational data sharing across industries. While prior research predominantly focused on technological functionality, we consider the relationship between business models and organizational roles and how their interplay enables sustainable value creation. We propose a conceptual framework to design the interplay between organizational roles and business models within a data ecosystem based on Gaia-X standards. Drawing on three use cases of Gaia-X 4 Advanced Mobility Services, we integrated empirical insights from semi-structured interviews. We defined independently organizational roles and business models at ecosystemic and firm-centric levels and assessed their compatibility. The conceptual framework shows how systematic assessment and iterative adjustments of organizational roles and business models can identify and minimize critical misalignments for a sustainable data ecosystem. Our findings guide scholars, practitioners, and policymakers in designing governance structures and business models for data spaces and ecosystems.

Specifications Table

*[Instruction for Specifications Table in comment box]*

**Table:** 

Subject:	European Data Spaces
Specific subject area	*Interplay of organizational roles and business models in data spaces based on the Gaia-X 4 Advanced Mobility Services research project*
Type of data	*Text version of semi-structured interviews*
Data source location	Country: Germany
Related research article	*None*

## 1 Introduction

In this era, digital technologies have transformed business processes and the value creation of digital products and services [[1], [2], [3]]. In this context, the technologies have led to exponential growth in the generation and sharing of data, which organizations use as a basis to create new data-driven business models [4,5]. Inter-organizational data-sharing relations are facilitated through the collaboration of various organizations in socio-technical networks known as “data ecosystems”, which aim to create shared value based on data exchange and the integration of diverse data-related capabilities. The term “data ecosystem” is not well established in academia and practice, nor is there a consensus on a general definition. Some authors, such as Oliveira et al. (2019), define data ecosystems as "socio-technical complex networks in which actors interact and collaborate with each other to find, archive, publish, consume, or reuse data as well as to foster innovation, create value, and support new businesses” [1]. Many organizations have significant concerns about sharing their data due to uncertainties in usage control and the potential risks of compromising their competitive position [6,7]. These concerns are addressed by the principle of data sovereignty, which empowers individuals and organizations to retain control over how their data is accessed, used, and shared [8]. Data sovereignty is facilitated through decentralized data spaces - a digital infrastructure that enables interoperable, secure, and sovereign data exchange.

The concept of data space is a key pillar of the European data strategy, which aims “to create a single European data space” [9]. This initiative led to several data space initiatives, such as Catena-X for the automotive supply chain and Manufacturing-X for the industry value chain, all compliant with the Gaia-X framework, aligning with European values. Until today, the International Data Spaces Association has listed 145 data spaces from 18 sectors [10]. The EU's Data Act supports these data-sharing initiatives by strengthening data sovereignty through mandatory obligations for companies to provide fair access to device-generated data for users and third parties [11]. Achieving compliance with the Data Act represents a substantial challenge for companies, particularly those in traditional industries, where data sharing has historically not been a central aspect of their business activities. However, the potential benefits from value creation facilitated by data ecosystems are expected to outweigh these challenges, offering new growth opportunities and fostering sustained competitiveness. Through its data strategy, Europe aims to position itself as a global leader in the data-driven world, projecting a value of €829 billion from the data economy by 2025 [12]). In research, decentralized ecosystems represent a novel concept that requires further investigation to gain a deeper understanding [13,14]. This understanding is required not only at the technological level but also at the organizational level of such ecosystemic structures. As previously mentioned, the primary aim of a data ecosystem is to create shared value, which is made possible through the business models that operate within the data ecosystem. However, companies continue to struggle when creating new business models for data ecosystems. One challenge is the necessity to evaluate business models from the perspective of the entire value network [15], which is governed by organizational roles in decentralized data ecosystems [[16], [70]]. This points out how governance and business models within data ecosystems are inherently intertwined.

Firstly, a network of business models that fails to deliver value to essential roles in the data ecosystem or concentrates critical roles in a single partner can threaten the sustainability of the ecosystem. The exit of such a partner could result in the absence of a crucial role in the ecosystem, such as a federator or data space infrastructure provider. Secondly, power imbalances can create tensions among participants, where dominant organizations may exploit their positions, undermining the principle of data sovereignty [17]. This can lead to mistrust and reluctance to share valuable data, ultimately stifling the potential benefits of collaborative data-sharing initiatives.

Moreover, the absence of equitable governance structures may hinder the establishment of fair data access and usage agreements, further exacerbating concerns around sovereignty. Addressing these issues is essential for establishing sustainable and inclusive data ecosystems where all participants can contribute to shared value creation.

Many studies have investigated decentralized data ecosystems regarding ecosystem design [18,19], organizational roles [16,[20], [70]], governance [21], value creation [6], business models [22] and their payment flows [23] in decentralized ecosystems. However, this was always done from an isolated perspective. Only a few authors have investigated the correlation between governance aspects and business models. For example, D’Hauwers et al. (2022) analyze the relationship between value creation and control across four dimensions: value proposition, value capture, value network, and data governance [24]. However, neither research nor practice has investigated how organizational roles and business models interact within a decentralized data space infrastructure.

This paper provides a comprehensive conceptual framework for aligning business models and organizational roles within a decentralized data infrastructure. It addresses this research goal by building on our research over the past three years on data space creation, business models, coordination, and governance challenges within the Gaia-X 4 AMS (Advanced Mobility Services) research project.

## 2 Background

### 2.1 Decentralized data ecosystems

Over the last two decades, the concept “ecosystem” has become relevant for research and practice in business strategy [25]. According to Teece (2014), “the concept of ecosystem might now substitute for the industry as a useful domain for performing economic analysis” [26]. The term ecosystem, derived initially from a biological metaphor, describes the co-evolution of interdependent species, particularly in response to fundamental changes [27]. This concept was adapted to the business context as a “business ecosystem”, which is an “economic community supported by a foundation of interacting organizations and individuals” [28] and became popular through Moore’s (1996) article. Until today, different definitions and research streams for ecosystem research have emerged. Adner (2017) provides an overview of different ecosystem perspectives and how these relate to established theories and concepts [25]. Jacobides et al. (2018) define an ecosystem as “a set of actors with varying degrees of multilateral, nongeneric complementarities that are not fully hierarchically controlled” [29]. Other definitions focus on value generation through an ecosystem structure, which is defined as “the alignment structure of the multilateral set of partners that need to interact in order for a focal value proposition to materialize” [25]. Ecosystem competition and innovation are interconnected at different organizational levels with different degrees of openness, which impacts the participants' collaborative or competitive behaviors [30]. Thereby, an ecosystem encompasses multiple dependent subsystems, which operate to varying degrees independently [31]. Such subsystems within data ecosystems are called “data spaces” (DS). Following Möller et al. (2024), data ecosystems are “socio-technical systems that emerge around one or multiple (federated) data spaces” [18]. DSs use decentralized architectures to ensure data sovereignty, which is implemented organizationally and technically to enhance sovereign data sharing across diverse organizations [19]. A prerequisite of decentralization is modularity, which fosters the development of new platforms by minimizing integration costs [31]. However, DS do not naturally emerge on their own. Their technical architecture and governance structure is usally governed by an institution or a consortium of institutions coordinated by an operating entity, which may also act as "data intermediary" when allowing the trading of data through data brokers or data marketplaces. A data intermediary serves as a “mediator between those who wish to make their data available and those who seek to leverage that data” [32]. In DS, data intermediaries mediate data sharing among institutions through a decentralized technical architecture, which employs certified software components called “data space connectors.” These connectors ensure data sovereignty through implemented usage policies [33].

In contrast to centralized data storage, DS enables data and service owners to retain control over the data and services they provide [34]. Also, the governance structure is decentralized through a consortium of institutions collectively administering the data space’s governance [19,35].

### 2.2 Business models and organizational roles within data spaces

The concept of business models originated approximately 50 years ago, focusing on information technology and aiming to model business systems [36]. However, due to the multidisciplinary components that constitute a business model [37], this concept has evolved across various disciplines [38]. Numerous frameworks have been proposed in the literature, highlighting a diverse set of constructs that serve as the foundation for business model composition [39]. Nevertheless, value creation is typically regarded as the central construct of a business model.

Traditional approaches to developing business models often adopt a “firm-centric” perspective on value creation, where value is proposed for a customer segment, and the value creator's business system is optimized [40]. However, this perspective was switched by Teece (2010), who defines a business model as the “design or architecture of the value creation, delivery, and capture mechanisms” of a business [41]. Following this architecture-oriented definition, other authors, such as Zott and Amit (2010), started understanding a business model as “a system of interdependent activities that transcends the focal firm and spans its boundaries” [42] - i.e. as an ecosystem per se. However, those perspectives remain “firm-centric” to ensure that companies “appropriate a share of the value created themselves” [42].

Intensive digitalization processes have led to innovative business models within the context of data ecosystems, where a sole “firm-centric” perspective of value generation may result in lower resilience and viability for the ecosystem. Similarly to contexts of industrial symbiosis [43], where companies establish a cooperative ecosystem to share resources based on their waste and by-products, data ecosystems also require an ecosystemic conception of the value creation, delivery, and capture mechanisms [15]. However, the ecosystemic conception of business models is, per se, a challenge.

Furthermore, creating and allocating value increases in complexity proportionally to the number of actors in an ecosystem [44], as it necessitates value co-creation among different partners within the ecosystem [24]. Additionally, unlike other typical business ecosystems, data ecosystems introduce the added complexity of enabling data recombination, which may lead to entirely new value propositions [45].

Other challenges in developing business models within data ecosystems arise from various perspectives. A common issue is data governance on an inter-organizational level, with many players [24,31].

The ecosystemic conception of value creation and delivery is also influenced by power asymmetries within the ecosystem, as companies with greater economic power may enforce policies or demand more protection for their data resources than what is feasible for other players [17]. Even if a fair network is established, the openness of the ecosystem to new entrants may threaten the power balance, especially if these new entrants can join the ecosystem too easily [46].

Given this delicate and complex balance, governance and business models must be developed in tandem to establish a data ecosystem that ensures resilience, fairness, and economic sustainability.

In this context, well-defined organizational roles are pivotal in translating governance principles into practical structures, ensuring coherent technical design, and facilitating seamless integration among diverse participants [47,48]. Within data ecosystems, clearly defined organizational roles support interoperability and trust and address the inherent complexities of aligning stakeholder incentives and objectives across varied institutional and technological boundaries [19,49]. Recent research emphasizes primary functions as data providers, service providers, application developers, and infrastructure providers, which create the ecosystem’s core value proposition [50,51]. Complementing these functions, additional organizational roles such as identity providers, data controllers, and specialized intermediaries foster trust and ensure compliance and interoperability, all critical for sustainable and resilient DSs [52]. These insights resonate with established organizational and role theory principles, which assert that clearly defined role expectations enhance coordination, mitigate uncertainty, and improve overall performance [53,54]. Unclear or conflicting organizational roles often obstruct collaboration and decision-making, which is challenging, especially for new digital collaborations as data ecosystems. Referring to Mintzberg (1973), who identified distinct managerial roles, underscores the importance of structured roles in navigating dynamic organizational landscapes [55].

By emphasizing activities and responsibilities that ecosystem actors overtake, such as ensuring data quality, managing interoperability, or orchestrating cross-organizational data sharing, ecosystems can react quickly to shifting market conditions, evolving regulatory frameworks, and technological advancements. This role-oriented perspective aligns with broader conceptualizations of digital ecosystems, wherein modular and reconfigurable role structures facilitate continuous innovation and the co-creation of value [25,29,56].

In summary, while business models outline how data creates value, organizational roles define the core responsibilities for keeping the data ecosystem (and, consequently, the business models within it) operational. Organizational roles specify how participants in the ecosystem contribute to the implementation of business models, ensuring alignment with strategic objectives. As business models evolve based on changes in data and market needs, roles shift and expand to adapt to these new business models. Therefore, these two concepts are deeply intertwined and must be considered in combination.

### 2.3 Gaia-X and advanced mobility services

Gaia-X is a dataspace initiative that seeks to provide a federated, secure, and open-source infrastructure for decentralized data ecosystems, thereby strengthening digital sovereignty of data owners [[57], [58], [59], [60]]. Gaia-X enables the trustworthy availability, sharing, and utilization of data and services. People, institutions, or devices can manage their digital identities and the associated login information (such as membership cards, certificates, or self-descriptions) independently without using a conventional central identity management system. Gaia-X-4-AMS (Advanced Mobility Services) is a research project of the GAIA-X 4 Future Mobility project family and aims to implement innovative and safety-critical mobility concepts based on the GAIA-X data and services ecosystem [61]. It contributes to data and service provision related to automated driving and cooperative system networks based on networked and automated vehicles and intelligent traffic infrastructures. This results in numerous advantages, such as the uncomplicated, cross-domain exchange of mobility data and reduced access barriers. To participate in the data space, an onboarding process must be completed to validate and trust new participants. On- and offboarding processes are ideally carried out by a third party (for example, a government instance) to ensure the stability of this role. In the case of a governmental solution, functionality instead of economic efficiency would perform the priority of this role. A subsidized or cost-bearing mechanism to prevent participant exclusion can significantly contribute to the stability and inclusiveness of the data space. In response to this need, the Gaia-X Digital Clearing House (GXDCH) concept was introduced [62]. The GXDCH is a platform for automated verification against Gaia-X standards, enabling participants to achieve compliance efficiently and transparently [62]. The Gaia-X Framework specifies the necessary technological functionalities, requirements, and software components to achieve Gaia-X compliance [62].

## 3 Research Design

The study's research setting is advanced mobility services within the German mobility sector, which is facing a meaningful shift to data-driven business models. Technology firms that have entered the mobility sector with new business models are driving this change. Significantly, the Big Tech companies have made enormous efforts to gain market shares. Furthermore, the EU-Data Act obligates firms along the value chain to disclose internal data, which may lead to new business models realized through data ecosystems [2]. The investigated ecosystem initiative is the research project Gaia-X 4 Advanced Mobility Services, which aims to implement a data ecosystem for mobility services in the context of autonomous driving. This implementation aims to be Gaia-X compliant and encompasses three prominent use cases used as a basis for the research described in this paper. The investigated use cases are described in Table 1 [61]. The study is built on a design science research approach [71] . The identifying artifacts should help academia and practice design data ecosystems with co-evolving organizational roles and business models in the context of the mobility sector.

**Table 1:** List of investigated cases of Gaia-X 4 AMS.

Use Case	Description
**ODD-Compatible Routing**	Operational Design Domain (ODD) is the operational environment in which an autonomous car can drive reliably and automatically, encompassing weather conditions, route speed, and road conditions. This use case aims to create a data ecosystem encompassing an advanced service capable of generating optimal routes based on the ODD conditions (i.e., the best route where the autonomous car can be entirely driven in an autonomous modus) and all the data and services required to run this service.
**Connected Rescue Corridor**	This use case involves building a data ecosystem that exchanges data between fire departments, traffic management, and autonomous cars to form a rescue lane and prioritize signalized intersections.
**Drone Provision Service**	This use case aims to create an ecosystem that connects several drone providers and fire departments to enable the use of drones in rescue and firefighting scenarios. The drones are provided as a service for firefighters, who can get image data and add other added-value services through the data ecosystem, such as artificial intelligence analysis of the images.

### 3.1 Case study research

We applied an exploratory case study approach to develop the conceptual framework for creating sustainable business models and organizational roles within data ecosystems. This methodological approach is suitable for qualitative data collection to investigate new phenomena not fully described by theories in the field [4,5]. The investigated use cases are described in Table 1. In each case, the research process was divided into three steps: “Define organizational roles”, “Define business models definition”, and “Assess cohesion”. Each step of the research process is described in detail in the following subsections.

#### 3.1.1 Define organizational roles

The organizational roles were based on the framework proposed by Gessler et al. (2025) [70], which was initially derived from the Institutional Role Model (IRM) [63]. The framework was adjusted and detailed through 16 interviews with experienced experts from the Gaia-X 4 Advanced Mobility Services research project. By allowing both structured and open-ended inquiry, the interview process accommodated context-specific insights and previously unconsidered roles [[64], [65], [66]]. The details about the qualitative data collection are provided in Table 2. The role evaluation was performed in a 5-hour workshop with 6 Research representatives and 8 Industry experts, who were asked to assess the final relevance of the roles and describe them adequately for the context of the ecosystem. The mapping of the meta-roles was also evaluated by analyzing shared characteristics of different organizational roles to build a cluster.

**Table 2:** Overview of data collection and participants for role definition.

Topic	Form	Participants	Duration
**Role Definition** • Identification of technological roles • Identification of economic roles • Use case allocation	16 semi-structured interviews	• 8 Research representatives (Research assistants and Postdoctoral researcher) • 8 Industry representatives (CEO, Manager hardware, Manager IT Consulting, Software engineer, Senior researchers)	Each interview lasting between 15 and 55 minutes
**Role Evaluation** • Relevance of roles • Role descriptions • Meta role mapping	1 Workshop	• 6 Research representatives (Research assistants and Postdoctoral researcher) • 8 Industry representatives (CEO, Manager hardware, Manager IT Consulting, Software engineer, Senior researchers, Software Developer)	05:00 h
**Role Assignment** • Evaluation of firms capabilities • Identification of suitable firms • Assigning roles to firms	3 workshops	• 6 Research representatives (Research assistants and Postdoctoral researcher) • 9 Industry representatives (CEO, Manager hardware, Manager IT Consulting, IT Consultant, Software engineer, Senior researchers, Software Developer)	Each lasting 01:30 h

#### 3.1.2 Define business models

The business models for each use case were defined iteratively, starting with an economic view to understand the interrelations among the business models. This approach was informed by [67], which describes data cooperation as an ecosystem. This definition was developed through interviews, workshops, and focus groups, which are summarized in Table 3 and explained below.

• **Initial Analysis:** We comprehensively analyzed each use case to understand consumer needs. One key project partner is the fire department of a German city, with two proposed solutions targeting them as primary consumers. Interviews with fire department representatives gathered insights into their requirements, including expectations regarding pricing, payment frequency, and other relevant details. These requirements were subsequently reviewed with data and service providers to align consumer expectations with provider capabilities.
• **Business Models Ecosystem Design:** A workshop was organized with all ecosystem providers, categorized into three groups based on their use cases. Participants were instructed to discuss how the value network of data, services, and infrastructure should interconnect to deliver collective value, and the value expected from the leading consumer. This discussion focused on business relationships since the technical interfaces had already been defined in prior flow diagrams. The workshop resulted in an initial sketch of the ecosystem, fostering collaboration among participants to align their business models with the data ecosystem's objectives.
• **Firm-Centric Business Model Design**: Following the ecosystemic analysis, we detailed a firm-centric view of each business model using the traditional design approach proposed by Osterwalder and Pigneur (2010). The individual business models were described, focusing on the following key building blocks:

○ **Value Proposition:** What value does their offering bring to the ecosystem?
○ **Customer Segment**: Are they providing their solution to another provider within the ecosystem or directly to the end consumer?
○ **Revenue Model:** What is their solution's most effective monetization strategy, considering all stakeholders' profitability and the ecosystem's sustainability?
○ **Cost Structures:** What data, service, and infrastructure offerings will they need to leverage within the ecosystem?
○ **Key Partnerships:** What agreements are necessary to ensure the effective operation of the ecosystem?

**Table 3:** Overview of data collection and participants for business model definition.

Topic	Form	Participants	Duration
**Initial analysis** • Identification of customer needs • Elicitation of the customer requirements	1 semi-structured interview	• 1 Representative of the Fire Department	90 Minutes
**Business Model Ecosystem** **Design** • Core data and service offerings • Possible revenue models • Interrelations among the business models	1 Workshop	• 6 Research representatives (Research assistants and Postdoctoral researcher) • 8 Industry representatives (CEO, Manager hardware, Manager IT Consulting, Software engineer, Senior researchers, Software Developer)	02:00 h
**Firm-centric Business Model** **design** • Detailing of business model building blocks: value proposition, customer segment, revenue model, cost structure, and key partnerships	3 co-working sessions	• 3 Industry representatives (CEO, Manager hardware, Manager IT Consulting)	Each lasting 01:30 h

The definition of the firm-centric business models was very diverse. Some partners required co-working sessions, where the definition was performed alongside the authors of this paper. Due to data confidentiality, some partners defined the remaining building blocks independently. Finally, some participants did not match the required expertise profile to provide further definitions of the business model since they had a technical (Software development) background. In these cases, the authors of this paper suggested the business model details based on best practices and references from the literature, according to the context of the use cases within the project.

#### 3.1.3 Assess cohesion

After both roles and business models were defined both from an ecosystemic and firm-centric perspective, we conducted focus groups with participants from each use case, aiming at assessing the cohesion between the business model definitions and the organizational roles definition, i.e., ensuring that the organizational roles of a participant company and the requirements of the business model they intend to operate are aligned and compatible Table 4. These discussions highlighted weaknesses and challenges in the ecosystemic conception based on the firm-centric business model definitions and incompatibilities between business model and role definitions.

**Table 4:** Overview of data collection and participants for cohesion assessment.

Topic	Form	Participants	Duration
**Focus groups** • Verification of cohesion between the firm-centric and ecosystemic definitions of the business models • Verification of cohesion between the firm-centric and ecosystemic definitions of the organizational roles • Verification of cohesion between the business model and organizational roles definition	3 Focus groups	• 6 Research representatives (Research assistants and Postdoctoral researcher) • 8 Industry representatives (CEO, Manager hardware, Manager IT Consulting, Software engineer, Senior researchers, Software Developer)	90 Minutes

## 4 Results

**Fig. 1:**
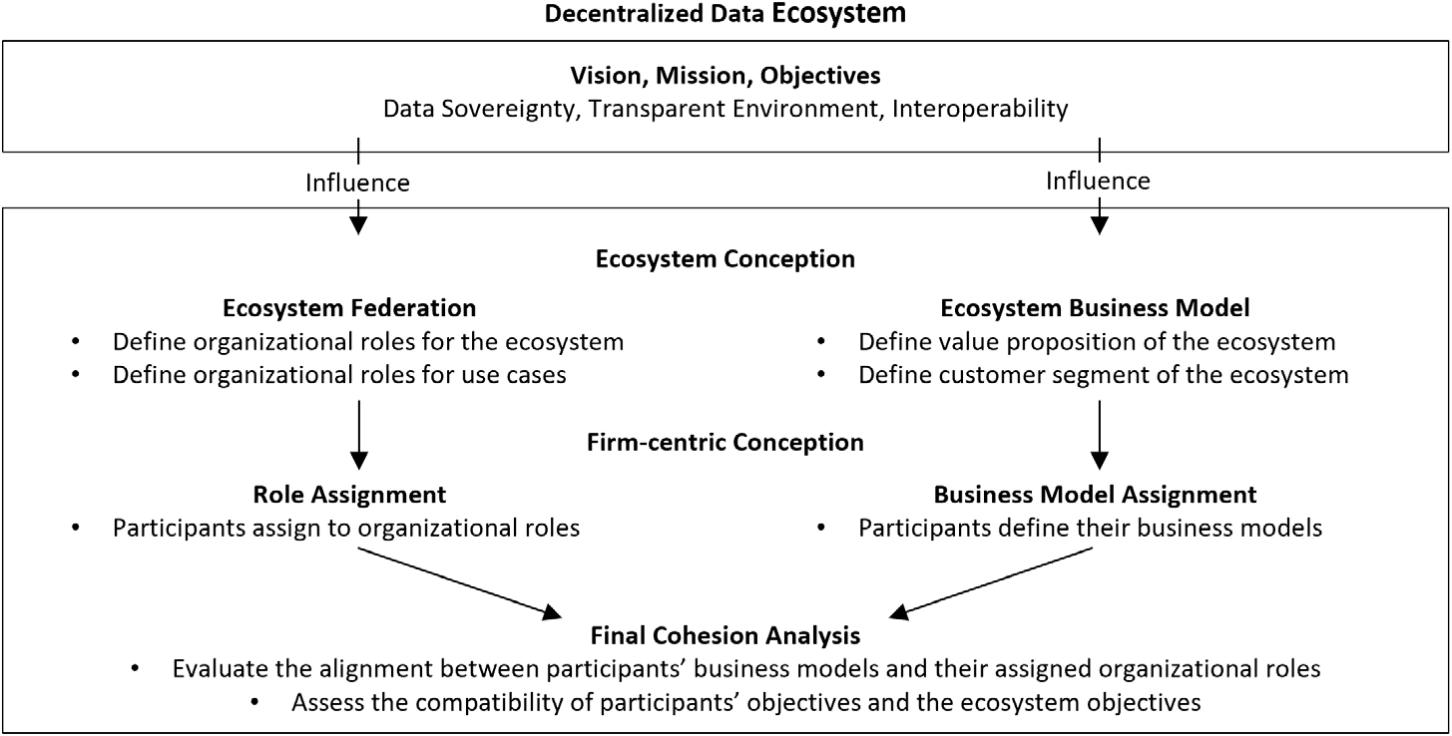
Conceptual framework for co-evolving role models and business models in a data ecosystem.

Based on the findings of the organizational roles and business models in each use case, we have defined a conceptual framework that incorporates insights to enhance a data ecosystem's resilience and sustainability (Fig. 1). Our findings emphasize the critical interplay between organizational roles and business models in decentralized data ecosystems. By analyzing the compatibility of organizational roles and business models, we recognized the need to develop a conceptual framework applicable to other contexts. At the top of the framework for decentralized data ecosystems should be a vision, mission, and objectives, which influence the organizational roles and business models of the ecosystem architecture and use cases. This contains values of data sovereignty, transparent environment, and interoperability. We developed the framework building on three different layers:

### 4.1 Ecosystem conception

The first part of the framework encompasses the ecosystemic conception regarding the business models and the organizational roles. Under the business model perspective, a core definition for the ecosystem is the value propositions it should provide, which orientate the participants on their business model definitions. Therefore, specific customer segments of the ecosystem and their needs and requirements must also be defined. Another part of this layer defines the necessary organizational roles required to fulfill the primary activities of the ecosystem. Additionally, the use-case-specific organizational roles must be defined along with the ecosystem participants. The use-case-specific organizational roles support activities and change over the ecosystem lifecycle when use cases are redefined or new use cases are integrated into the ecosystem.

### 4.2 Firm-centric conception

Based on the ecosystemic definition of the expected value proposition and organizational roles, each participant must structure how their business model will fit the ecosystem value proposition and which responsibilities should be assigned to them.

At this layer, the ecosystem participants play a pivotal role in defining their business models within the ecosystem. They structure a firm-centric definition of their business models while ensuring alignment with the ecosystemic value proposition. The definition of the business model follows the approach proposed by Osterwalder and Pigneur (2010) [37] but with some special considerations. Participants need to take into consideration that the building blocks “value proposition”, “customer segment”, “revenue stream”, “cost structures”, and “key partnerships” need to be defined in alignment with the overall definition of the ecosystem, considering their interfaces with other participants and ensuring that those building blocks establish a good trade-off between the partners involved in the use case. The remaining building blocks (i.e., “customer relationship”, “customer channels” and “key resources”) can be defined following the traditional approach proposed by Osterwalder and Pigneur (2010) [37]. Additionally, the organizational roles are assigned to the participants based on their capabilities, resources, and willingness to take on a role. This ensures the efficiency of the organizational roles. Here, it is necessary to consider the centralization of critical roles and potential power asymmetries. Critical organizational roles— i.e., roles that, if their responsible party were to leave the ecosystem, could generate instability or even jeopardize the ecosystem's viability—must be assigned with a well-considered balancing strategy. This strategy, which may involve the establishment of redundancies, ensures that responsibilities are distributed among multiple participants to mitigate risks associated with the loss of a single individual. Furthermore, ensuring that no single participant is responsible for multiple critical roles is crucial, as this could create vulnerabilities and increase dependency on specific individuals. By thoughtfully distributing these roles and responsibilities, organizations can enhance stability, resilience, and continuity within the ecosystem, safeguarding its overall functionality and sustainability.

### 4.3 Final cohesion analysis

The last layer assesses with the participants the alignment between their independently defined business models and their assigned organizational roles. Specifically, in this step, it is necessary to assess whether the roles assigned to each company include all necessary functions to implement the proposed business model successfully. Furthermore, it is necessary to examine whether these roles collectively support the operation of the entire ecosystem from the business model perspective. This is essential for uncovering inconsistencies that reduce the ecosystem's efficiency and functionality, such as missing roles or incompatibilities between assigned roles and the effective business model of each partner. Solutions aligning with the participants and the ecosystem’s objectives will address the inconsistencies. These solutions can encompass redefining or defining new organizational roles and adjusting business models.

## 5 Application

This chapter presents the findings of the applied conceptual framework on the cases of Gaia-X 4 Advanced Mobility Services. Our findings underscore the critical interplay between organizational roles and business models as co-evolving determinants of resilience, trust, and sustainability within decentralized data ecosystems. Assessing the compatibility between organizational roles and business models provides deeper insights into the conditions necessary for stable, strategically aligned, and value-generating ecosystems.

### 5.1 Ecosystem conception

The ecosystem conception layer encompasses the independent parts of ecosystem federation and ecosystem business models described in the following.

#### 5.1.1 Ecosystem federation (ecosystemic roles definition)

**Fig. 2:**
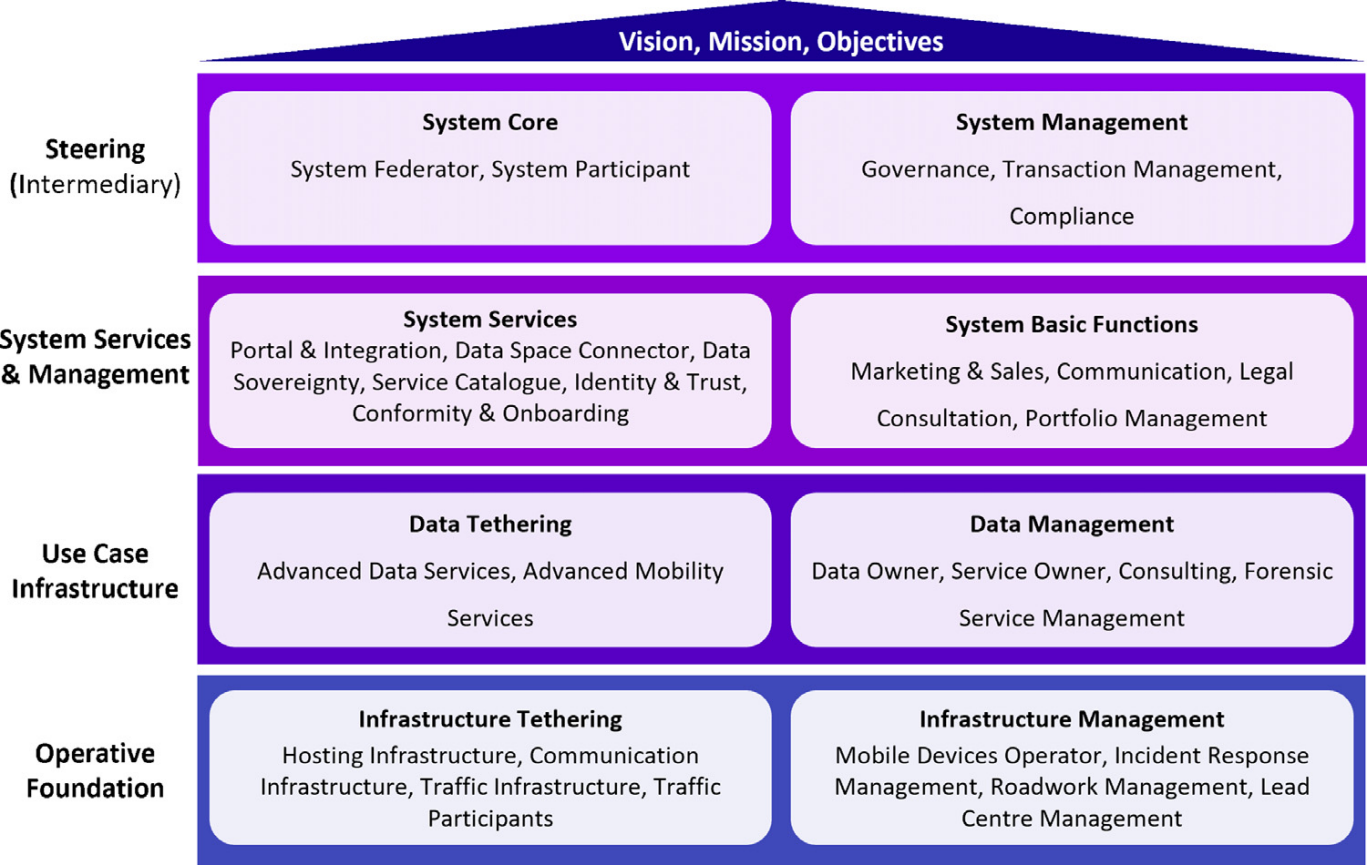
Organizational roles of the Gaia-X 4 advanced mobility services ecosystem.

We defined 32 organizational roles, which were grouped into eight meta-roles, each contributing to different layers of the ecosystem (Fig. 2). The meta-roles all support the overall vision, mission, and objectives, which form the foundation and basis for the functionality and reliability of the data ecosystem and are presented in the following [[16], [70]]:

#### 5.1.2 Ecosystem perspective of the business models

**Fig. 3:**
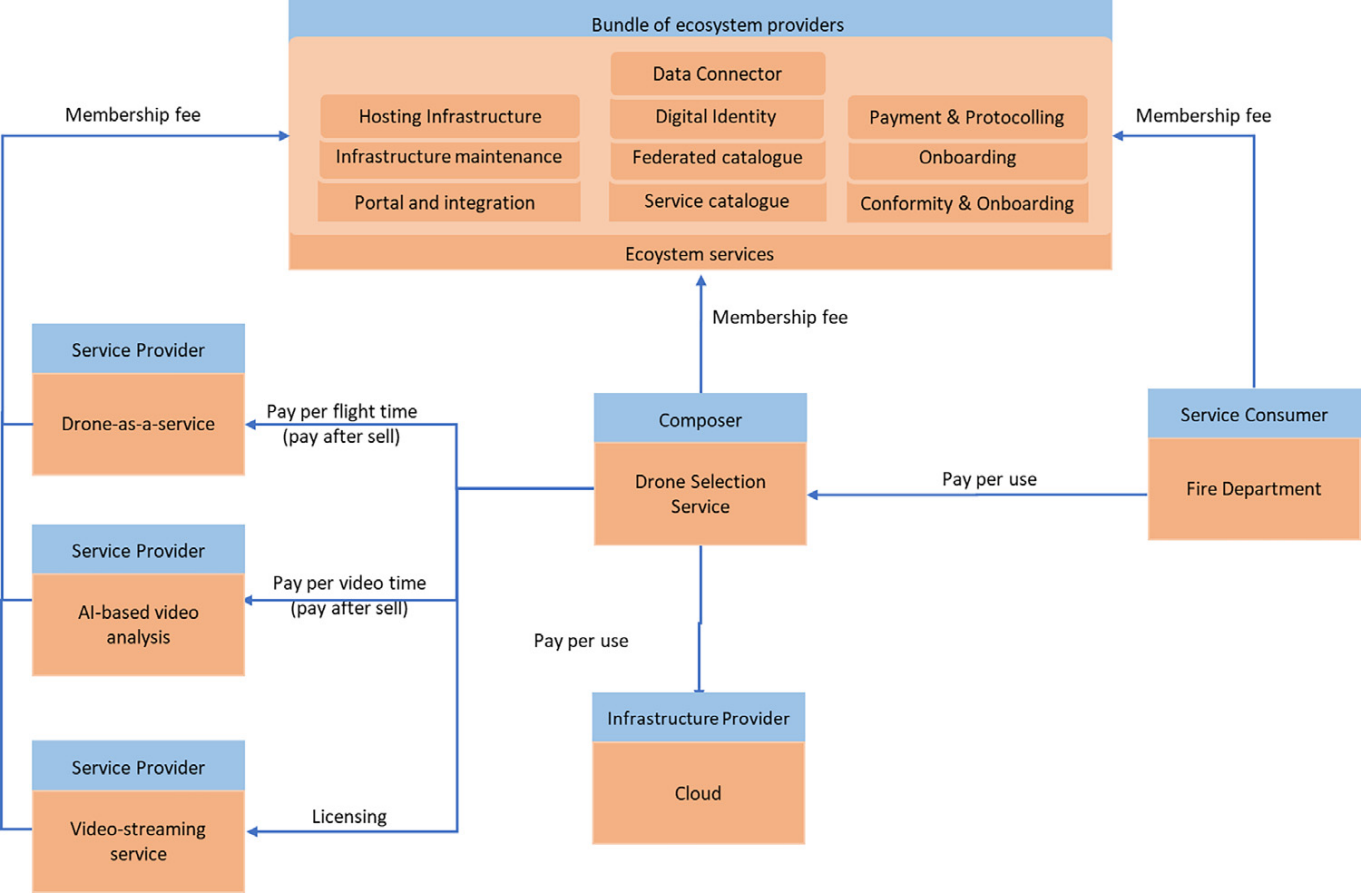
Anonymized example of the value network for the use case ”Drone Provision Service”.

The business model's ecosystem perspective was defined separately for each use case. One example of a business model network originating from one of the use cases is presented for clarity, anonymized, and exemplified in Fig. 3. In the figure, the arrows illustrate the financial flows and the revenue models associated with one of the use cases: the Drone Provision Service. The orange rectangles depict the data or service offering encompassed by the business model's value proposition, and the blue rectangles depict a generic description of the partner responsible for providing that business model.

*Service Consumer:* The end user of the data or service provided within an ecosystem. This consumer does not utilize the data or service to create further solutions for the ecosystem; instead, they are the final consumer. In the example shown in Fig. 3, the fire department acts as the Service Consumer, compensating the provider for the Drone Provision Service. This participant represents the Customer Segment that the ecosystem targets.

*Data/Service Providers:* These are the providers of data or service offerings that are not sold directly to the end consumer. Instead, these offerings are utilized to create the final solution delivered to the end consumer. In Fig. 3, the data and service offerings include AI-powered video analysis algorithms, a direct video-streaming service between the drone provider and the fire department, and the drone-as-a-service itself. Each provider flexibly selects revenue models to ensure a financially resilient data ecosystem, including purchase, pay-per-use, and licensing-specific algorithms or datasets. However, the most appropriate solution appears to come from implementing those revenue models in a pay-after-sale structure,whereby the composer can utilize the data and service offerings from Data and Service Providers to create the solution but will only pay for those offerings after each purchase made by the Service Consumer. A pay-after-sale structure ensures risk sharing among all participants, including the Composer, and stimulates the participation of more Composers since their risk is significantly reduced.

*Infrastructure Providers* are the participants who supply the necessary infrastructure to enable the use case. The example depicted in Fig. 3 includes providers of cloud storage solutions for video footage. The most suitable revenue model identified for these providers is pay-per-use solutions, which significantly reduce the cost of each service unit sold to the Service Consumer.

The *Composer* acts as an orchestrator who integrates various data, service, and infrastructure offerings from the ecosystem to deliver a final solution or service bundle for the service consumer. They also serve as a financial intermediary, invoicing the Service Consumer and managing payments to the other data, service, and infrastructure providers involved in the use case. In the example illustrated in Fig. 3, the composer offers a drone selection interface that enables the fire department to enter mission descriptions and select based on these inputs. These services will make up the final service bundle. This bundle includes the most suitable drone for the requested mission, AI-powered video analysis algorithms, a direct video-streaming service between the drone provider and the fire department, and a cloud storage solution for the video footage. The revenue models utilized by the composer are highly flexible. However, the most appropriate ones are typically pay-per-use solutions, which help reduce the overall financial risk for the other participants.

*Bundle of Ecosystem Providers*: The ecosystem providers supply all the essential infrastructure for the data space and ensure that the data ecosystem remains functional. To promote cohesion and integration and reduce the number of invoices generated for each participant, ecosystem providers should operate as a bundle, charging participants appropriately to cover the ecosystem's costs. Two revenue models appear to be most suitable for bundles of ecosystem providers. To cover fixed costs, membership fees could be implemented; however, membership fees for Service Consumers and Data/Service Providers are recommended to be lower than those charged over Composers. This approach increases the availability of data and service offerings. It enhances the diversity of Service Consumers, making the ecosystem more attractive to potential Composers and fostering the growth of data ecosystems in their early stages. As a second revenue model, it is proposed that the Bundle of Ecosystem Providers charge the Composer a commission on each financial transaction to cover transaction costs. While the resilience of the ecosystem is likely to be greater with a combination of both revenue models, it is also feasible to operate using only one of them.

### 5.2 Firm-centric conception

The firm-centric conception layer encompasses the independent parts of role assignment and business model assignment described in the following.

#### 5.2.1 Role assignment

The role assignment was based on the defined organizational roles derived from the ecosystem federation layer. Therefore, three focus group meetings, each lasting 90 minutes, were applied. The three focus group meetings included the same six research representatives and the same nine industry representatives. The focus group meetings were conducted in three steps. First, the participants were asked to evaluate how suitable their institution is for each defined organizational role in terms of capabilities, resources, and supporting company targets. Second, the most suitable institutions were discussed and identified. The third step encompassed aligning the organizational roles to the most suitable ones. The authors are not allowed to publish the names of those who are aligned. However, aligned institutions were described based on their value proposition to present the results of the role assignment (see Table 5). As shown in Table 5, not all organizational roles were aligned to an institution of the data ecosystem. The reasons, therefore, are incomplete “ecosystem blueprints”. To create an ecosystem the federator defines an “ecosystem blueprint”, which the participants agree [7], and defines the vision and core value proposition of the ecosystem [8]. The problem, which scholars also recognize, is the incompleteness of the “ecosystem blueprints” because they do not encompass the potential of new technologies [9], which can impact the ecosystem's value proposition [7].

**Table 5:** Overview of the role assignment.

Metarole	Role	Aligned Institution
**System Core**	System Federator	IT Service Provider
	System Participant	All Participants
**System Services Provider**	Portal & Integration	IT Service Provider
	Dataspace Connector	IT Service Provider
	Data Sovereignty	IT Service Provider, Research Institute, Software Provider
	Service Catalogue	IT Service Provider
	Identity & Trust	Not aligned with any partner
	Conformity and Onboarding	IT Service Provider, Research Institute
**Data Tethering**	Advanced Data Services	Engineering Office
	Advanced Mobility Services	Research Institute / IT Consulting
**Infrastructure Tethering**	Hosting Infrastructure	Technology Provider, IT Service Provider
	Communication Infrastructure	Technology Provider
	Traffic Infrastructure	Alignment to an external partner
**System Basic Functions**	Marketing & Sales	Alignment to an external partner
	Communication	Alignment to an external partner
	Legal Consultation	Alignment to an external partner
	Portfolio Management	IT Service Provider
**Data Management**	Data Owner	Research Institute, Engineering Office
	Consulting	Research Institute
	Service Owner	Research Institute, Engineering Office, Software Provider
	Forensic Service Management	Alignment to an external partner
	Mapping Management	Software Provider
**Operations Management**	Mobile Device Operator	Aircraft Systems Provider
	Incident Response Management	Fire Brigade
	Roadwork Management	Alignment to an external partner
	Lead Centre Management	IT Service Provider

#### 5.2.2 Business model assignment

As described in the methodology, the firm-centric business models were designed following very distinct strategies depending on the partner's expertise regarding the business model theme and their willingness to define their business models collaboratively.

The firm-centric definition of the business models was essential to define the activities each participant company should cover in detail. At this moment, for example, some discussions arose about the viability of allowing the customers to flexibly build bundles of services to be consumed through the catalog of the data space, which was identified as unfeasible by many participants. Therefore, during the firm-centric definition of the business model, the role of a “composer” arose as a necessity in defining the “Partnerships” building block within the business model. This, of course, would lead to further modifications in the Ecosystemic view of the business models.

Furthermore, even with the delimitations of viable business models for the ecosystem composition, many other firm-centric decisions and requirements generated impacts that could unbalance the pre-defined ecosystem. One example is the requirement raised by the fire department that payments should be processed yearly due to their current complex payment processes. Even though this requirement was elicited at the beginning of the whole process, it only became relevant once the definition of the firm-centric business model was approached. There, it was noticed that by accepting this kind of payment flow, costs would be incurred over specific partners (like the Drone-as-a-service provider and the provider of AI-based video analysis) without generating revenues for an extended time. In this case, this decision impacted the ecosystem once again, requiring the addition of a money broker to facilitate the transactions and serving as an accelerator for the data and service providers to have access to their payments quickly. Those imbalances and impacts are discussed in the following section.

### 5.3 Final cohesion analysis

During the final cohesion analysis, we identified misalignments discussed with the ecosystem participants in iterative workshops.

Besides some examples that were already discussed in the previous subsection, one illustrative example emerged in the context of the drone provision service (see Fig. 3). While the firm-centric business model conceptualization identified a “composer” entity responsible for orchestrating service offerings and structuring the financial flows between consumer and all the providers involved, this role was not considered during the definition of organizational roles. From the perspective of role definition, interview participants viewed payment and monetization management as non-core functions of the data ecosystem. They proposed that individual ecosystem participants could handle these functions directly or rely on external solutions rather than integrating complex revenue flows within the ecosystem’s architecture. However, during the sessions in which organizational roles and business models were compared, the participants pointed out that creating advanced services by combining data and service offerings would be implausible to be accomplished by relying on the data consumers for combining the offerings. Instead, a "Composer" (like discussed in the previous section) would be necessary to ensure that advanced services (or combined bundles of data and service offerings) are offered and financial flows are structured.

Another interesting finding involved the notion of a bundle of ecosystem providers conceived as a single orchestrating participant in the business model perspective. In contrast, the predefined roles framework differentiated between the system federator and the meta-role system services, each operating at distinct ecosystem layers. While a service composer and a bundle orchestrator may enhance efficiency, reduce transaction costs, and ensure consumers' access to advanced services, their centralizing influence raises concerns about dependency and organizational concentration. Another risk is if such a bundle of ecosystem providers were to employ revenue models based on commission rates since it could threaten partners' sovereignty over transaction data, as sales volumes and financial transactions between data/service providers and data/service consumers would need to be shared with the bundle of ecosystem providers.

The problem toward centralization became evident during the role assignment process, notably as larger corporations demonstrated the capacity to assume multiple critical roles simultaneously. By covering a combination of roles from the meta-roles system core, system services, and system management, these actors could potentially consolidate influence and reshape the balance of the ecosystem. Recognizing and mitigating these centralization tendencies and potential power asymmetries is thus essential for maintaining a resilient and sustainable decentralized data ecosystem.

### 5.4 Discussion and conclusion

With this study, we contribute to the growing research of data ecosystems by providing a conceptual framework for designing the interplay between organizational roles and business models within a data ecosystem based on Gaia-X standards. This work enriches prior work on data ecosystem design, governance, and value creation [19,22,49]. Our findings show that organizational roles and business models within data ecosystems are not static but dynamically interdependent constructs. This is consistent with literature that views ecosystems as interlinked constellations of actors, each with evolving roles and responsibilities that collectively shape value creation and capture [25,29]. Organizational roles emerge and transform in response to ongoing adjustments in business models and strategic positioning, underscoring the adaptive nature of data ecosystems.

The iterative approach of defining organizational roles independently from business models and subsequently conducting a systematic assessment of their compatibility proved instrumental in identifying and minimizing critical misalignments. Mitigating these frictions necessitates a reassessment of assigned organizational roles and adjusting revenue models. Institutions must recognize business models as dynamic capabilities rather than static constructs to navigate evolving markets and technological landscapes effectively [68]. Due to their cooperative structure, especially data ecosystems, their participants must continually reassess the interaction of organizational roles and business models to identify adjustments to improve efficiency and stay competitive over the lifecycle. However, the interplay between business models and organizational roles needs to be assessed iteratively from an ecosystem and a firm-centric perspective to ensure an ecosystem and a firm-centric view. Further cohesion analysis regarding roles and business models also enables reassessing the power imbalances that may arise in an ecosystemic arrangement. From a governance perspective, the interplay between roles and business models aligns closely with principles of institutional economics and complexity management frameworks, which emphasize the reliance of ecosystems on adaptive institutional logic and coordination mechanisms [69]. A sustainable data ecosystem depends on the participants’ willingness and resources to realign organizational roles to maintain strategic fit, economic viability, and compliance with ecosystem standards [25,29].

### 5.5 Limitations and future research

Our study’s insights into the interplay of organizational roles and business models are derived exclusively from the Gaia-X 4 AMS research project, which may limit their applicability to other contexts. Future research extends the conceptual framework to other industries and regions, such as the manufacturing or energy sector, to elaborate on identified roles, and the framework can be generalized or require contextual adaptations. Furthermore, the research project investigated does not cover all lifecycle stages until maturity. Analyzing the development of organizational roles and business models and associated key performance indicators over time would enrich understanding of the interplay between organizational roles and business models and their impact on governance structures and business model adaption.

In this digital era with exponential data growth, the sustainability and competitiveness of data ecosystems depend on the interplay between well-defined organizational roles and business models. Our study’s conceptual framework and empirical insights enrich the research on data ecosystems.

## CRediT Author Statement

**Jens Gessler:** Conceptualization, Methodology, Investigation, Data curation, Writing – original draft, Visualization, Project administration. **Maiara Rosa Cencic:** Conceptualization, Methodology, Investigation, Data curation, Writing – original draft, Visualization, Project administration. **Christian Metzner:** Investigation, Data curation, Writing – original draft, Project administration **Wolfgang H. Schulz**: Writing – review & editing, Supervision. **Horst Wieker**: Writing – review & editing, Supervision. **Kai Lindow:** Writing – review & editing.

## Data Availability

it's a conceptual paper. Further data analysing data can be provided if paper is relevant (Reference data)
